# Editorial: Resistance to third-generation tetracyclines

**DOI:** 10.3389/fmicb.2022.1114660

**Published:** 2023-01-12

**Authors:** Eun-Jeong Yoon, Seok Hoon Jeong, Rustam Aminov

**Affiliations:** ^1^Korea National Institute of Health, Korea Disease Control and Prevention Agency, Cheongju-si, Republic of Korea; ^2^Department of Laboratory Medicine, Research Institute of Bacterial Resistance, Yonsei University College of Medicine, Seoul, Republic of Korea; ^3^The School of Medicine, Medical Sciences and Nutrition, University of Aberdeen, Aberdeen, United Kingdom

**Keywords:** third-generation tetracyclines, glycylcyclines, fluorocyclines, animals, environment, One Health

Discovery and development of many antimicrobials have followed the common route that begins from the isolation and characterization of an antimicrobial agent from a natural source, and then the original molecule is modified through several iterations to broaden the spectrum of pathogens targeted, improve its pharmacokinetic and pharmacodynamic properties, and, most importantly, to overcome bacterial antimicrobial resistance (AMR), which inevitably emerges and propagates among bacterial pathogens (Aminov, 2017). In this respect, the tetracycline family of antimicrobials is not an exception. The first natural tetracyclines were discovered in the 1940s and shortly thereafter introduced into the clinical and agricultural practices. They demonstrated excellent efficacy against a broad range of bacterial pathogens, with little side effects, and thus were widely used in human and veterinary medicine as well as in many areas of agriculture for metaphylaxis and growth-promoting purposes. The growing resistance problem, however, prompted the development of the second generation tetracyclines such as doxycycline and minocycline in the 1960s. Once again, their efficacy was compromised by bacterial resistance and the development of the third-generation of tetracyclines (3GT), glycylcyclines and fluorocyclines, was commenced in the late 1990s. The currently available drugs of this generation include glycylcycline, eravacycline, omadacycline, and sarecycline, which were approved in the mid-2000 to the late 2010s.

In the beginning of the antimicrobial era, there have been almost no monitoring efforts directed at detection and analysis of AMR. Thus, we have a very limited understanding of the processes in the past that led to the acquisition and spread of resistance to “older” antimicrobials. The introduction of novel antimicrobials such as the 3GT offers unique opportunities in this regard and may allow to discern the mechanisms by which bacteria become resistant. Besides, developments in genomics and metagenomics allow rapid and large-scale analyses of bacterial samples of various origin carrying suspected AMR genes and mobile genetic elements (MGEs) associated with them. Concerning resistance toward 3GT, the (meta)genomic data available at the time suggested that one of the most likely mechanism of resistance to emerge could be *via* the acquisition and dissemination of *tet*(X) genes, which encode flavin-containing monooxygenases that are capable of degrading 3GT (Aminov, 2009, 2013). One of the contributing factors to this process could be the use of “older” tetracyclines in agriculture, which selects for *tet*(X) as well (Aminov, 2021).

Indeed, as demonstrated by papers in this Research Topic, microbiota from agricultural settings display a range of the *tet*(X) genes that are located on MGEs such as plasmids. Wang J. et al. isolated 49 *Escherichia coli* strains from pigs, and six of them were resistantt toward tigecycline. The resistance was encoded by the *tet*(X4) gene, which was located on a IncFIA18/IncFIB(K)/IncX1 hybrid plasmid. The *tet*(X4)-carrying *E. coli* ST761 lineage seems frequent in different areas in China, with a high risk of further dissemination. As demonstrated by Li et al., Enterobacterales (*Citrobacter* spp., *E. coli, Enterobacter hormaechei*, and *Providencia alcalifaciens*) and *Acinetobacter* spp. (*A. variabilis, A. lwoffii*, and *A. baumannii)* isolates from chicken farms also carry a range of the *tet*(X) genes, with the dominance of *tet*(X4). Within the extended One Health framework, Chen et al. analyzed plasmid-encoded diversity of the *tet*(X) genes in *Acinetobacter* spp. isolates of different origin, ranging from humans to agricultural animals to migratory birds and the environment. GR31 group of plasmids, which carried different variants of *tet*(X), seems to be prevalent among *Acinetobacter* spp. isolates. Thus, within the One Health context, the extensive diversity of mobile *tet*(X) variants in different ecological compartments should be considered as a risk factor and measures have to be taken to reduce this risk.

Implementation of potential risk-reduction strategies, however, requires a better understanding of underlying biological processes that drive the selection and maintenance of resistance to 3GT. The most obvious factor is the selection of resistance to 3GT by “older” tetracyclines (Aminov, 2021). Once selected, however, the fitness cost of carrying a *tet*(X)-containing IncFII plasmid by a bacterial host could be substantial (Xiao et al.). Location of *tet*(X) on other plasmids may ameliorate this cost though. For example, the mechanism of stable maintenance of the *tet*(X) genes in bacterial populations could be aided by their location on IncX1 plasmids, since these plasmids encode a histone-like nucleoid-structuring protein, which reduces the fitness cost of plasmid carriage by bacterial hosts and contributes to the stable plasmid inheritance (Cai et al.).

Other mechanisms of resistance toward 3GT are present in clinical isolates, and these are mainly mediated by efflux pumps. As demonstrated by Hao et al., the drug efflux mechanism is implicated in tigecycline resistance in a clinical *Klebsiella pneumoniae* isolate. Wang Y. et al. established that in other species of *Klebsiella* such as *K. variicola, K. quasipneumoniae* and *K. michiganensis*, resistance to tygecycline is mediated by resistance-nodulation-division- (RND) type efflux pumps (TMexCD2-TOprJ2 clusters) and these are located on IncHI1B type plasmids. The RND-type efflux pump-mediated tigecycline resistance also operates in *A. pittii* (Ding et al.). In *Staphylococcus aureus* isolates, resistance to 3GT is mainly conferredby mutations in the genes encoding MepRAB efflux pumps and 30S ribosomal subunits, while overexpression of other efflux pump genes such as *tet*(38), *tet*(K) and *tet*(L) is also noted in several tigecycline-resistant strains (Zeng et al.). Thus the current mechanisms of resistance to 3GT in clinical isolates is *via* the mutations that up-regulate the expression of efflux pumps. These mechanisms are presumably generated during the therapy, and they are species-specific, which makes the dissemination to various pathogens unlikely.

Presently, plasmid-encoded *tet*(X) and especially the *tet*(X4) variant are widely distributed in various ecological compartments in China as demonstrated by papers in this Research Topic as well as by publications elsewhere (Sun et al., 2019; Li et al., 2021; Feng et al., 2022; Zhang et al., 2022). Geographical boundaries for plasmid-encoded *tet*(X4) seems to be expanding with its detection in Pakistan (Mohsin et al., 2021) and Turkey (Kürekci et al., 2022). Moreover, plasmid-encoded *tet*(X4) seems to be emerging in clinical settings (Zhai et al., 2022). These concerning developments may pose a significant risk for public health and must be dealt with promptly.

## Author contributions

All authors listed have made a substantial, direct, and intellectual contribution to the work and approved it for publication.
